# Changes in Eating Behaviours among Czech Children and Adolescents from 2002 to 2014 (HBSC Study)

**DOI:** 10.3390/ijerph121215028

**Published:** 2015-12-15

**Authors:** Jaroslava Voráčová, Erik Sigmund, Dagmar Sigmundová, Michal Kalman

**Affiliations:** Institute of Active Lifestyle, Faculty of Physical Culture, Palacký University Olomouc, Tr. Miru 117, Olomouc 77111, Czech Republic; erik.sigmund@upol.cz (E.S.); dagmar.sigmundova@upol.cz (D.S.); michal.kalman@upol.cz (M.K.)

**Keywords:** changes, eating habits, eating behaviours, children, adolescents, HBSC, Czech Republic

## Abstract

Many children skip breakfast, consume soft drinks/sweets and do not eat the recommended amounts of fruit and vegetables. Poor eating habits in children tend to be carried over into adulthood. The changes in eating behaviours of Czech 11-, 13- and 15-year-old children were examined by frequency of breakfast (on weekdays and weekends), fruit, vegetable, sweet and soft drink consumption using data obtained from the Health Behaviour in School-aged Children (HBSC) surveys in 2002, 2006, 2010 and 2014. Logistic regression was used to analyze changes in eating behaviours. The findings showed a significant increase (only in girls, *p* ≤ 0.001) in prevalence of breakfast consumption (on weekdays) and a decrease in daily consumption of soft drinks (in boys and girls, *p* ≤ 0.001), sweets (in boys and girls, *p* ≤ 0.01) and fruit (in boys, *p* ≤ 0.01; in girls, *p* ≤ 0.001) between 2002 and 2014. Daily vegetable and breakfast on weekends consumption remained statistically unchanged over time. More frequent daily fruit, vegetable and breakfast (on weekends) consumption was reported by girls and younger children, whereas daily soft drink intake was more prevalent in boys and older children. There is a need for re-evaluation of current policies and new initiatives to improve the eating habits of Czech children.

## 1. Introduction

An unhealthy diet is one of the major risk factors for chronic diseases [1,2,3] that are the leading causes of death globally [1]. Recent evidence shows that even though the prevalence of non-communicable diseases is not very common among youth, there is early onset of risk behaviours in adolescents [4,5]. Healthy eating habits such as high fruit and vegetable intake, reduced soft drink consumption and eating breakfast regularly are the key elements to prevent chronic disease and promote health [6,7,8]. Several studies have shown that health risk behaviours such as eating habits established in childhood and adolescence tend to be maintained into adulthood [9,10,11,12,13]. 

Recent longitudinal data also indicate associations between unhealthy dietary patterns during adolescence and the prevalence of obesity in adulthood [14,15,16]. Overweight and obesity in childhood and adolescence is linked to premature mortality and physical morbidity in adulthood [17]. Positive associations are documented between increased intake of sugar-sweetened beverages [18,19,20,21], irregular breakfast eating [19,22], inadequate consumption of fruit and vegetables [23], high fat food intake [21] and development of overweight and obesity in youth. Furthermore, diets high in fat and sugar lead to higher intake of energy and lower intakes in protein, polyunsaturated fat, fiber, β-carotene equivalents, folate, vitamin C, zinc, and potassium [24]. Implementing effective, sustainable dietary interventions such as school-based education programs have been proven as a possible solution to reduction in body weight [25], as well as improvement of overall health and well-being. Prior to implementing such programs it is crucial to first identify the current situation and trends and patterns in the eating behaviours of youth.

In the Czech Republic and other European countries, dietary trends have changed over the past decade [4,5,26,27]. There is also wide variation in eating habits across countries [28,29] that may be attributable to cultural practices and socioeconomic factors [4] as well as different national policies and practices at schools [30]. Data from longitudinal studies monitoring dietary patterns in children and adolescents over time have demonstrated deterioration in regular consumption of breakfast [31,32], fruit and vegetables, as well as intake of sweetened beverages [32]. In addition, evidence using cluster analysis among children showed that, while children do change their diet, they are more likely to follow the same eating pattern (healthy or unhealthy) at a later age [12]. The evidence suggests that girls consume more vegetables and less sweetened drinks compared to boys [12,32]. Furthermore, several cross-sectional studies across the world have evaluated long-term changes in eating habits in children and adolescents. An increase in daily fruit and vegetable intake between 2002 and 2010 was found in the majority of countries of Europe and North America [5,29,33]. Based on the current findings, girls and younger children were more likely to consume fruit and vegetables than boys and older children [29]. In addition, in most Western countries, children and adolescents are reported to eat far less than the recommended amounts of fruit and vegetables [28]. Patterns and trends in sweet-beverage and soft drink consumption also differ across countries. Decreased consumptions of soft drinks were reported in Norway [34], Scotland [33], the USA [35] and the Netherlands [36], however, an increased consumptions were found in Lithuania [3] and Ireland [37]. Older children and boys reported consuming soft drinks more frequently [5]. To our knowledge there is limited number of cross-sectional studies that have monitored trends in breakfast consumption during weekdays [31,33,38,39,40,41] and only one study addressed changes on weekends [31]. Current data suggests that even though there might be slight improvements in breakfast eating in some countries [41], others show a decline [31,40] and the breakfast consumption prevalence remains low among children and adolescents [4]. Boys and younger children tend to eat breakfast more frequently [4]. In addition breakfast skipping was reported to be more frequent on weekend days than on weekdays [31]. School intervention programs can be used as an effective tool to improve breakfast eating [38]. 

Even though a few studies have addressed the dietary pattern trends among schoolchildren, none of them monitored all six eating behaviours (breakfast on weekdays and weekends, fruit, vegetable, sweet and soft drink consumption) over an extended period. Monitoring trends in dietary habits is crucial for planning future intervention programs and the formulation and evaluation of national policies and nutritional guidelines [42]. The eating habits of Czech children and adolescents seem to be worse or similar than of those in other European countries [4]. In the Czech Republic, the data on trends in overweight/obesity, physical activity and screen time among children and adolescents is known [43], however, we do not have a good understanding of the changes in eating behaviours and their impact on youth’s health and well-being. Also, little is known about these trends in relation to gender and age. Therefore, the aim of this study was to provide an insight into dietary changes among Czech children and adolescents aged 11–15 years in relation to their age and gender between 2002 and 2014. The changes in eating habits were examined by frequency of breakfast, fruit, vegetable, sweet and soft drink consumption using data obtained from the Health Behaviour in School-aged Children (HBSC) study.

## 2. Methods

The data from a Czech cross-sectional survey of the HBSC study collected in 2002, 2006, 2010 and 2014 was used. The HBSC research study is done in collaboration with the WHO Regional Office for Europe [4] and is currently administered in 43 countries and regions across Europe and North America [44]. The HBSC collects data every four years to provide a better understanding of health behaviours, health and well-being in the social context of 11-, 13- and 15-year old children and adolescents [42]. This study followed the standardized international research protocol and procedures developed by the HBSC Research Network members [42].

### 2.1. Survey Design and Sample

Data were collected through self-completed questionnaires administered in the classroom. Participants were 11-, 13- and 15-year-old boys and girls attending 5th, 7th and 9th grades of primary school. Stratified cluster sampling by region and district was used to select the participants and the primary sampling unit was the school class. Only students at state-funded schools were included in the study. Questionnaires were administrated in the school classroom by the principal or deputy in each of the participating schools. Participants were provided with a verbal description of the study and were given the chance to ask questions prior to participation. The questionnaire was anonymous. Only participants who provided the consent forms signed by their parents/guardians were included into the study. Upon completion of the data collection, all files were exported to the HBSC International Data Bank at the University of Bergen for cleaning and compiling into an international data set [42]. Data were then returned to the Czech Republic for further statistical processing. Response rates were between 75% and 85% in all four years. The number of participants eligible for the analysis was 4058 in 2002, 4149 in 2006, 3950 in 2010 and 4380 in 2014.

During each year of the study, the survey was carried out in the spring, between April and June. All students participated on a strictly volunteer basis and all data collected were confidential. Only the investigators had access to the data, which were coded with ID numbers to remove any identifying information about the participants. The Ethical Committee of Palacký University in Olomouc approved the study prior to administration of the surveys (No. 17/2013). 

### 2.2. HBSC Questionnaire

The questionnaire contained a wide range of questions assessing participants’ health and health behaviours, social background, family relations, eating habits, *etc.* [42]. In this study only questions pertaining to eating behaviours were used. The source language of all questions was English. The questionnaire was translated into Czech and then back translated by the study coordinator/administrator. The same questions were used each year of the study.

### 2.3. Eating Behaviours

Five eating behaviours (consumption of breakfast, fruit, vegetables, soft drinks and sweets) were assessed by the frequency of weekly consumption. Breakfast consumption was assessed by two questions relating to weekdays and weekends: “How often do you usually have breakfast (more than a glass of milk or fruit juice)?” The response options for weekdays were “I never have breakfast during weekdays/one day/two days/three days/four days/five days” and for weekends “I never have breakfast during the weekend/I usually have breakfast on only one day of the weekend (Saturday or Sunday)/I usually have breakfast on both weekend days (Saturday and Sunday)”. Regular breakfast eaters were students who reported consuming breakfast on five weekdays and on both days on the weekend. Consumption of fruit, vegetables, soft drinks and sweets was assessed by the question “How many times a week do you consume fruit/vegetables/sweetened soft drinks/sweets?” with response options “never/less than once a week/two to four times a week/five to six times a week/once a day/more than once a day”. Regular consumers were students who reported consuming each item at least every day or on more than once a day.

### 2.4. Statistical Analysis

All statistical analyses were processed in IBM SPSS v. 19.0 (IBM, Armonk, NY, USA). Frequencies of consumption of breakfast, fruit, vegetables, soft drinks and sweets were calculated for both gender and age categories for each year of the study. Descriptive data was calculated for each variable for all survey years (Table 1). Changes in eating behaviours were analyzed by using logistic regression (Enter method) analyses for each eating behavior and gender and age, with survey year 2002 as reference group. Likelihood of daily consumption of each behaviour (regular daily consumers) for each gender and age was described by Odds Ratios (ORs) with 95% confidence intervals (CIs), where boys were the reference group for gender and 11 years for age. All variables set at *p* < 0.05 were considered statistically significant. 

**Table 1:** Descriptive characteristics of the samples, health behaviour in school-aged children study, Czech Republic 2002–2014.

	2002		2006		2010		2014	
	Boys	Girls	Boys	Girls	Boys	Girls	Boys	Girls
n=	(1918)	(2140)	(2099)	(2050)	(1885)	(2065)	(2127)	(2253)
	%	%	%	%	%	%	%	%
**Age category ^§^**								
11 years	34.3	33.6	32.0	32.0	34.2	30.7	30.5	31.6
13 years	32.0	33.6	33.7	33.5	31.2	34.8	33.6	34.3
15 years	33.7	32.8	34.3	34.5	34.6	34.5	35.9	34.1
**Gender **	47.3	52.7	50.6	49.4	47.7	52.3	48.6	51.4
Eating behaviours *								
Daily soft drinks	30.4	26.1	34.3	27.1	24.6	19.9	17.2	14.2
Daily sweets	25.5	24.9	30.1	29.9	26.5	28.2	21.0	21.5
Daily fruit	37.0	49.0	33.1	45.2	37.3	47.1	33.1	41.8
Daily vegetables	23.4	31.1	23.8	31.6	26.0	37.6	24.2	31.3
Daily breakfast (weekday)	58.0	44.7	51.7	45.0	57.6	49.5	60.2	54.3
Daily breakfast (weekend)	84.9	86.1	82.8	85.2	80.7	85.1	83.1	87.2

Notes: ^§^ 11 years (11.00–11.99 years, 5th grade), 13 years (13.00–13.99 years, 7th grade), 15 years (15.00–15.99 years, 9th grade); * Expressed in percentage of participants who perform the eating behaviour at least daily.

## 3. Results

The descriptive characteristics of the participants are shown in Table 1. The HBSC questionnaire was completed by a total of 19,931 children: 4998 in 2002, 4751 in 2006, 4409 in 2010 and 5773 in 2014 (data not shown). After removing subject that did not meet the age requirements, total sample eligible for analysis was 16,537 children and adolescents of age 11 years (32.4%), 13 years (33.3%) and 15 years (34.3) (Table 1).

### 3.1. Overall Changes in Eating Behaviours

From 2002 to 2014, the data showed an improvement in the percentages of daily consumers of sweets, soft drinks and breakfast during weekdays among Czech children and adolescents. On the contrary, the findings also indicated a decline in daily intake of fruit and no significant change in daily vegetable consumption and breakfast eating on weekends. Overall, reported consumption of sweets, soft drinks and fruit decreased significantly by 15% (*p* < 0.01), 45% (*p* < 0.001) and 13% (*p* < 0.01), respectively, and breakfast consumption on weekdays increased by 11% (*p* < 0.01) over the time period (data not shown). 

### 3.2. Gender and Age Specific Changes in Eating Behaviours 

Disparities were found in changes of dietary patterns by gender and age over the 12-year period. Changes in the prevalence of six eating behaviours according to age and gender are shown in Table 2.

**Table 2:** Changes in prevalence of six eating behaviours in Czech children and adolescents between 2002–2014.

	2002	2006	2010	2014	2014 *vs.* 2002		
Odds Ratio to Reach the Variables ^1−6^	% ^a^	% ^a^	% ^a^	% ^a^	OR	95% CI	
						Lower	Upper
**Daily soft drinks** ^1^							
Boys	30.4	34.3 ^∧^	24.6 ^♯^	17.2	0.48 ***	0.41	0.55
11 years	**27.9** ^♯^	35.0 ^O^	**18.7**	15.3	0.46 ***	0.35	0.61
13 years	29.0	34.3	26.6	18.9	057 ***	0.44	0.74
15 years	**34.2** ^∧^	33.8 ^O^	**28.5** ^O^	17.3	0.40 ***	0.31	0.52
Girls	26.1	27.1 ^∧^	19.9 ^♯^	14.2	0.47 ***	0.40	0.54
11 years	22.5 ^♯^	26.4 ^O^	**15.5**	13.2	0.52 ***	0.39	0.69
13 years	29.1	31.1	23.2	15.3	0.44 ***	0.34	0.57
15 years	26.8 ^∧^	23.9 ^O^	**20.9** ^O^	14.0	0.44 ***	0.34	0.58
**Daily sweets** ^2^							
Boys	25.5	30.1	26.5	21.0	0.79 **	0.67	0.90
11 years	**20.7** ^O^	30.7 ^♯^	**21.4**	22.4	1.11	0.85	1.44
13 years	27.4	28.3	27.9	21.8	0.74 *	0.57	0.95
15 years	**28.3** ^♯^	31.3	**30.3**	18.9	0.59 ***	0.46	0.76
Girls	24.9	29.9	28.2	21.5	0.83 **	0.72	0.95
11 years	**28.4** ^O^	**25.3** ^♯^	**21.6**	18.9	0.59 ***	0.46	0.75
13 years	23.0	32.6	32.8	24.0	1.06	0.83	1.34
15 years	**23.3** ^♯^	**31.8**	**30.1**	21.5	0.91	0.71	1.16
**Daily fruit** ^3^							
Boys	37.0 ^O^	33.1 ^O^	37.3 ^O^	33.1 ^O^	0.82 **	0.72	0.94
11 years	**42.9** ^O^	**37.6** ^O^	**42.2** ^O^	**40.8** ^O^	0.92	0.74	1.14
13 years	38.3 ^O^	36.5 ^O^	40.0 ^♯^	32.8 ^O^	0.78 *	0.63	0.98
15 years	**29.8** ^O^	**25.3** ^O^	**29.6** ^O^	**25.6** ^O^	0.81	0.64	1.02
Girls	49.0 ^O^	45.2 ^O^	47.1 ^O^	41.8 ^O^	0.74 ***	0.66	0.83
11 years	**54.8** ^O^	**48.4** ^O^	**54.7** ^O^	**50.4** ^O^	0.84	0.68	1.03
13 years	47.4 ^O^	46.4 ^O^	45.9 ^♯^	40.9 ^O^	0.77 *	0.63	0.94
15 years	**44.8** ^O^	**40.8** ^O^	**40.6** ^O^	**34.1** ^O^	0.64 ***	0.52	0.79
**Daily vegetables** ^4^							
Boys	23.4 ^∧^	23.8 ^O^	26.0 ^O^	24.2 ^∧^	1.03	0.89	1.19
11 years	**28.5** ^♯^	**25.9** ^∧^	**29.4** ^O^	**28.7** ^∧^	1.01	0.79	1.29
13 years	22.0 ^O^	26.1 ^∧^	27.3 ^♯^	23.9 ^∧^	1.11	0.86	1.44
15 years	**19.8** ^O^	**19.3** ^O^	**21.3** ^O^	**20.0** ^O^	1.02	0.78	1.32
Girls	31.1 ^∧^	31.6 ^O^	37.6 ^O^	31.3 ^∧^	1.00	0.88	1.14
11 years	**34.6** ^♯^	**33.9** ^∧^	**43.1** ^O^	**35.2** ^∧^	1.03	0.82	1.28
13 years	30.1 ^O^	32.8 ^∧^	33.5 ^♯^	29.6 ^∧^	0.98	0.78	1.22
15 years	**28.5** ^O^	**28.0** ^O^	**36.2** ^O^	**29.2** ^O^	1.03	0.82	1.30
**Daily breakfast (weekday)** ^5^							
Boys	58.0 ^O^	51.7 ^∧^	57.6 ^∧^	60.2 ^∧^	1.08	0.95	1.22
11 years	**63.7** ^O^	**55.8**	68.2 ^♯^	**67.8**	1.20	0.96	1.52
13 years	54.7 ^O^	54.2 ^♯^	58.4 ^O^	60.3 ^∧^	1.26 *	1.01	1.56
15 years	**55.5** ^O^	**45.2**	46.1	**52.6** ^O^	0.89	0.72	1.10
Girls	44.7 ^O^	45.0 ^∧^	49.5 ^∧^	54.3 ^∧^	1.45 ***	1.29	1.63
11 years	**54.7** ^O^	**51.6**	62.0 ^♯^	**66.5**	1.64 ***	1.32	2.04
13 years	42.8 ^O^	38.9 ^♯^	45.3 ^O^	52.9 ^∧^	1.50 ***	1.22	1.85
15 years	**36.5** ^O^	**44.4**	41.3	**43.6** ^O^	1.34 **	1.09	1.66
**Daily breakfast (weekend)** ^6^							
Boys	84.9	82.8	80.7 ^♯^	83.1 ^♯^	0.86	0.72	1.02
11 years	**88.3**	**84.7** ^O^	84.7 ^♯^	**85.7** ^O^	0.79	0.57	1.10
13 years	84.6	84.7	83.3	83.3 ^∧^	0.90	0.67	1.22
15 years	**81.8**	**79.1**	74.2 ^∧^	**80.2**	0.90	0.69	1.18
Girls	86.1	85.2	85.1 ^♯^	87.2 ^♯^	1.09	0.91	1.30
11 years	**91.1**	**90.7** ^O^	88.8 ^♯^	**92.4** ^O^	1.19	0.81	1.74
13 years	86.3	82.9	86.5	88.1 ^∧^	1.18	0.87	1.61
15 years	**80.8**	**81.9**	80.1 ^∧^	**81.2**	1.03	0.79	1.33

Notes: % ^a^: percentage of adolescents who performed the eating behaviour at least daily; ^1–6^ OR of daily consumption for each behaviour; logistic regression Enter method (LR): 2014 *vs.* 2002; OR: odds ratio (reference group is a cohort of 2002); CI: 95% confidence interval; * *p* < 0.05, ** *p* < 0.01, *** *p* < 0.001; Age difference—**bold**: significant difference between 11 and 15 year-old boys and girls for each survey year, at *p* < 0.05; Gender difference—significant difference between boys and girls of the same age ^♯^ *p* < 0.05, ^∧^ *p* < 0.01, ^O^ *p* < 0.001.

From 2002 to 2014, a significant decrease in prevalence over time was documented among both boys and girls in daily soft drink (*p* < 0.001), sweet (*p* < 0.01) and fruit (*p* < 0.01 for boys, *p* < 0.001 for girls) consumption (Table 2). In addition, significantly (*p* < 0.001) more girls, but not boys, reported eating breakfast on weekdays in 2014 compared to 2002.Changes in eating behaviours also varied by age of the students between 2002 and 2014 (Table 2). Significant (*p* < 0.001) changes in percentage of soft drink consumers were found among 11-, 13-, and 15-year-old boys and girls. A decrease in daily sweets intake was reported only among 13- and 15-year-old boys (*p* < 0.05, *p* < 0.001, respectively) and 11-year-old girls (*p* < 0.001) and change in daily fruit intake only among 13-year-old boys (*p* < 0.05) and 13- and 15-year-old girls (*p* < 0.05, *p* < 0.001, respectively). Breakfast eating (weekdays) improved significantly in all age groups in girls (*p* < 0.001, *p* < 0.001, *p* < 0.01, respectively) and only in 13-year-old boys (*p* < 0.05).

### 3.3. Gender and Age Differences

The results indicated significant variation in prevalence of some eating behaviours between boys and girls in each survey year as well as differences between younger children (11 years) and adolescents (15 years) (Table 2). As shown in Table 2, across all survey years, significantly more girls tended to report daily eating of fruit, vegetables and breakfast on weekends, compared to boys. In 2014, the gender difference for these eating behaviours was 9%, 7% and 4%, respectively. On the contrary, more boys reported consuming breakfast on weekdays, with the gender difference in prevalence being 6% in 2014. Even though boys were more likely to report consuming soft drinks the differences were not significant in most of the survey years. 

In 2014, significant differences between 11- and 15-year-olds were indicated in reported consumption of fruit (*p* < 0.001 both genders), vegetables (*p* < 0.001 boys, *p* < 0.01 girls) and breakfast on weekdays (*p* < 0.001 both genders) and on weekends (*p* < 0.01 boys, *p* < 0.001 girls) but not in soft drink and sweet intake (Table 2). In the most recent data, higher percentages of younger boys and girls consumed fruit, vegetables, and breakfast on weekdays and weekends, with difference for boys/girls being 15%/16%, 9%/6%, 15%/23% and 6%/11%, respectively. When comparing 2002 and 2014, age differences remained constant for consumption of fruit, vegetables and breakfast on weekdays and weekends but not for soft drinks and sweets intake. Only in 2002, but not 2014, significant age differences were indicated in consumption of soft drinks and sweets.

## 4. Discussion

This study addressed the changes in consumption of several eating behaviours among Czech children and adolescents from 2002 to 2014. Overall, the findings indicated improvement in some of the food habits (soft drinks, sweets, breakfast during weekday) and stagnation or diminishments in others (fruit, vegetables, breakfast on weekends) over time. The most significant changes were observed in reported percentages of daily consumers of soft drinks (decrease), sweets (decrease) and fruit (decrease) as well as breakfast on weekdays (increase). The data also confirmed that more girls and younger children tended to report eating fruit, vegetables and breakfast (on weekends) every day and the age and gender differences were significant in both 2002 and 2014. In addition, more frequent daily soft drink intake was reported by boys and older children, however, significant age and gender variations were seen only in 2002. The findings regarding age and gender variations were consistent with results from other countries [29]. Compared to younger children, worse food habits in adolescents could be explained by a higher degree of autonomy in this age, more freedom of decision making on purchasing food and eating/drinking outside the home more often [45]. 

Other studies that addressed dietary intakes in the Czech child population reported that the consumptions of fruit and vegetables do not meet the national and WHO recommendations [46,47]. The WHO and the Czech Ministry of Health recommend at least 400 g of fruit and vegetable daily, which is about 3–5 servings of vegetables (potatoes included) and 2–4 servings of fruit [28,47]. Jakubikova *et al.*, [47] documented that only 22% of Czech children aged 4–14 years consumed five or more servings of fruit and vegetables per day. Also, fruit was consumed almost twice as often as vegetables [47]. Low numbers of children consuming fruit (37.5%) and vegetables (27.8%) daily in 2014 and the failure to meet the set recommendations [46,47] emphasize the need for efforts to improve the food habits of children.

In spite of some improvements in the diet of Czech children during recent years, the Czech Republic still lags behind several European countries in the consumption of fruit and vegetables [4]. Between 2002 and 2010, the most pronounced positive changes in fruit intake (OR > 1.6) were found in Denmark, England, Norway, Ukraine, USA, Wales and Scotland [29]. A significant increase in vegetable consumption was found in Spain, Denmark, Hungary, England, Wales, Greece and Austria (OR > 1.6) [29]. More frequent consumption of fruit and vegetables in these countries could be explained by many factors such as the implementation of nation-wide policies, programmes and government strategies to improve the eating habits of young people as well as cultural differences and socioeconomic factors. For example, in 2007, the Norwegian government implemented the nation-wide free school fruit scheme in all secondary elementary schools (grades 8–10) and all combined schools (grades 1–10) in order to improve fruit and vegetable intake [48,49,50]. The evaluation of this programme showed long-term effects of the free school fruit programme that resulted in a great increase in students’ intake from 2001 to 2008 [48]. In addition, several countries such as the USA, Great Britain, Sweden and New Zealand have implemented breakfast clubs at school where free breakfast is provided in the morning prior to classes [51,52,53,54]. Regular participation in breakfast clubs was associated with improved school attendance, academic performance and diet quality (especially in families with low socioeconomic status) [51,52,53,54]. Scotland is another example of a country that implemented initiatives to establish lifelong healthy eating habits of their children. The Scottish Government developed new nutritional requirements for school meals and drinks via the Schools (Health Promotion and Nutrition) Act 2007 and in 2010 also introduced Curriculum for Excellence that included health and wellbeing and programmes such as free fruit initiatives and breakfast clubs [33]. Such as nation-wide initiatives and school-based interventions have been proven to have an important impact on eating habits of children via increasing the children’s and parents’ knowledge of the recommendations for fruit and vegetable intake [36,48,49,55]. 

Unfortunately, the Czech Republic has not implemented many nation-wide policies or guidelines regarding nutritional requirements for schools encouraging healthy eating habits of children that can be carried into adult life. However, some attempts to increase the knowledge of healthy eating have already been administered which might have contributed to improvement of some eating behaviours (Table 2). One of the most recent efforts of the Czech Ministry of Health is an adjustment of the Educational Act that regulates sales of unhealthy food and drinks in school vending machines and cafeterias [56]. This Act became effective in September 2015, however, the specific information on the “banned” food and drinks have not been published yet. Therefore, the significant improvement in percentage of daily consumers of soft drinks and sweets between 2002 and 2014 could be explained by promotional strategies of various non-government agencies and nutrition organizations that use marketing through social media (TV, newspaper, internet, Facebook, *etc.*) to affect school management [57,58,59]. Currently, it is within the competence of school principals to decide what food/drinks will be offered in vending machines or cafeterias. Another current effort of the Czech government is a school-based trial programme announced by the Ministry of Education, Youth and Sports in March 2013 [60]. This trial programme is targeted to make changes in current primary school curriculum in areas of physical activity and nutrition. The trial is currently being tested in several pilot schools and involves primary schools (grades 1–5), teachers and parents’ involvement. Monitoring trends of eating habits in Czech children and adolescents could be important tool for developing and optimizing such initiatives.

As shown in Table 2, the fluctuation could be observed in some of the eating behaviours (fruit and vegetables) over time. Between 2002 and 2006, percentage of daily fruit (vegetable) consumers decreased (did not changed, respectively) which was followed by an increase in 2010 and another decrease in 2014. These inconsistent trends in fruit and vegetable consumptions could be explained by the implementation of a “free fruit (renamed to free fruit and vegetable) at school programme” which is a nation-wide programme supported by European Union [61]. Students attending the participating primary schools (grades 1–5) are entitled to receive a free piece of fruit or vegetable while at school. This programme is to increase awareness of the importance of eating fruit and vegetables and to encourage better eating practices. Currently, more that 85% of Czech primary schools are participating in the project [61]. In the Czech Republic, the programme became effective in autumn 2009 which might be, together with more vigorous enforcement by the school staff at the early stages, the result of improvement in fruit and vegetable consumption in 2010. The downward trend observed in the following years might be due to the lower reinforcement of the programme as well as a low budget allocation per child per year (currently 333 Czech crowns) that does not cover the cost of providing a piece of fruit or vegetable every school day as in some other countries [48,62]. Czech students are entitled to receive free fruit or vegetables a minimum of twice per month and it is unclear whether this frequency will be enough to establish long-term positive changes in food habits. Also, according to yearly evaluations of the programme, fruit and vegetable provision was the most common during breaks between classes and the least common during the class that could result in inadequate motivation of children to consume provided fruit and vegetables [61]. 

## 5. Strengths and Limitations

The strengths of the present study are its use of a representative sample for the Czech Republic, including different age groups and genders, and using a standardized research protocol of a large cross-national study which data could be used for comparison with other countries in Europe and North America. However, there are some limitations that need to be considered. First, the use of a self-reported questionnaire could be a source for recall and social desirability bias, errors in self-observation and bias in trends due to on-going initiatives. Despite the potential for errors, the HBSC food frequency questionnaire (FFQ) has shown high test-retest reliability and validity compared to 24 h food behavior checklist (FBC) and a 7-day food diaries (FD) [63] as well as good to moderate agreement (percent agreement 0.70–0.87, kappa 0.43–0.65) between frequency and 7 times a 24-h recall measures for breakfast [64]. Fair agreement (percent agreement 0.53–0.84, kappa 0.26–0.54) was documented for lunch meal and different results were found for evening meal (high percent agreement 0.83–0.95 but poor kappa 0.14–0.19) [64]. Second, only frequencies of intakes were used to describe the eating behaviours. This data did not provide information on portion sizes, amounts of food consumed and whether children met the dietary recommendations. A 24-h recall food behaviour checklist might be the better way to collect this information [42]. Third, the data collected could be affected by seasonal bias. In the Czech Republic, the lowest likelihood of fruit consumption was indicated during the spring months [47,65], compared with October and December [47,65]. The lowest likelihood of soft drink intake was in January to February (compared with May to June). Since our survey was administered in the spring (April-June), the frequencies of fruit and vegetables could be underestimated. Fourth, the questionnaire related problems could be a source of another potential error [66]. Due to the limited space in the questionnaire, the food items (e.g., fruit, vegetables, sweetened soft drinks, breakfast, *etc.*) were not defined and could be misinterpreted. Last, but not least, the data was not adjusted for parental socio-economic status. Family affluence scale (FAS) that has been used to identify socioeconomic inequalities in adolescents in the HBSC studies was calculated in different way (questionnaire items) in 2002, 2006, 2010 and 2014. Therefore, it is difficult to analyze trends in the same dietary habits in view of different kind of calculated FAS scale. However, there is the lack of studies addressing this issue in the Czech Republic and future research should focus on describing current adolescent eating habits controlled for socio-economic status.

## 6. Conclusions

In summary, this study provides an insight into the current food consumption habits of Czech children and adolescents and describes the changes of these behaviours from 2002 to 2014. An improvement was found in consumptions of daily soft drinks and sweets for all ages and both genders (except 13-year old girls). However, fewer children reported eating fruit every day and there was almost no change in vegetable intake over time. Moreover, daily breakfast consumption on weekdays increased among all children (except 15-year old boys) but a significant change was reported only among girls and 13-year-old boys. In 2014, fewer boys but more girls reported eating breakfast on weekends compared to 2002. These findings indicate the need for a re-evaluation of current national policies, initiatives and nutritional requirements and regulations in schools along with health promotion strategies to improve diet of children at home-based and community settings. Future research should continue monitoring trends in the Czech Republic to further evaluate effectiveness of on-going changes in national policies and other intervention programmes. In addition, it would be useful to investigate dietary intakes including amounts of food items and the frequencies of consumptions.

## References

[1] World Health Organization (2011). Global Status Report on Noncommunicable Diseases 2010. http://www.who.int/nmh/publications/ncd_report2010/en/.

[2] Marmot M., Wilkinson R.G. (2006). Social Determinants of Health.

[3] Zaborskis A., Lagunaite R., Busha R., Lubiene J. (2012). Trend in eating habits among Lithuanian school-aged children in context of social inequality: Three cross-sectional surveys 2002, 2006 and 2010. BMC Public Health.

[4] Currie C., Morgan A., Currie D., de Looze M., Roberts C., Samdal O., Smith O.R.F., Barnekow V. (2012). Social Determinants of Health and Well-Being among Young People. Health Behaviour in School-Aged Children (HBSC) Study: International Report from the 2009/2010 Survey.

[5] Fismen A.S., Smith O.R., Torsheim T., Samdal O. (2014). A school based study of time trends in food habits and their relation to socio-economic status among Norwegian adolescents, 2001–2009. Int. J. Behav. Nutr. Phys. Activity.

[6] Affenito S.G. (2007). Breakfast: A missed opportunity. J. Am. Diet. Assoc..

[7] Bazzano L.A., Serdula M.K., Liu S. (2003). Dietary intake of fruits and vegetables and risk of cardiovascular disease. Curr. Atheroscler. Rep..

[8] Vartanian L.R., Schwartz M.B., Brownell K.D. (2007). Effects of soft drink consumption on nutrition and health: A systematic review and meta-analysis. Am. J. Public Health.

[9] Emmett P.M., Jones L.R. (2015). Diet, growth, and obesity development throughout childhood in the Avon longitudinal study of parents and children. Nutr. Rev..

[10] Mikkila V., Rasanen L., Raitakari O.T., Pietinen P., Viikari J. (2004). Longitudinal changes in diet from childhood into adulthood with respect to risk of cardiovascular diseases: The cardiovascular risk in young Finns study. Eur. J. Clin. Nutr..

[11] Nigg C.R., Amato K. (2015). The influence of health behaviors during childhood on adolescent health behaviors, health indicators, and academic outcomes among participants from Hawaii. Int. J. Behav. Med..

[12] Northstone K., Smith A.D., Newby P.K., Emmett P.M. (2013). Longitudinal comparisons of dietary patterns derived by cluster analysis in 7- to 13-year-old children. Br. J. Nutr..

[13] Pearson N., Salmon J., Campbell K., Crawford D., Timperio A. (2011). Tracking of children’s body-mass index, television viewing and dietary intake over five-years. Prev. Med..

[14] Johnson L., Mander A.P., Jones L.R., Emmett P.M., Jebb S.A. (2008). Energy-dense, low-fiber, high-fat dietary pattern is associated with increased fatness in childhood. Am. J. Clin. Nutr..

[15] Merten M.J., Williams A.L., Shriver L.H. (2009). Breakfast consumption in adolescence and young adulthood: Parental presence, community context, and obesity. J. Am. Diet. Assoc..

[16] Moore L.L., Bradlee M.L., Gao D., Singer M.R. (2006). Low dairy intake in early childhood predicts excess body fat gain. Obesity.

[17] Reilly J.J., Kelly J. (2011). Long-term impact of overweight and obesity in childhood and adolescence on morbidity and premature mortality in adulthood: Systematic review. Int. J. Obes..

[18] Fiorito L.M., Marini M., Francis L.A., Smiciklas-Wright H., Birch L.L. (2009). Beverage intake of girls at age 5 y predicts adiposity and weight status in childhood and adolescence. Am. J. Clin. Nutr..

[19] Laska M.N., Murray D.M., Lytle L.A., Harnack L.J. (2012). Longitudinal associations between key dietary behaviors and weight gain over time: Transitions through the adolescent years. Obesity.

[20] Lim L., Banwell C., Bain C., Banks E., Seubsman S.A., Kelly M., Yiengprugsawan V., Sleigh A. (2014). Sugar sweetened beverages and weight gain over 4 years in a Thai national cohort—A prospective analysis. PLoS ONE.

[21] Millar L., Rowland B., Nichols M., Swinburn B., Bennett C., Skouteris H., Allender S. (2014). Relationship between raised BMI and sugar sweetened beverage and high fat food consumption among children. Obesity.

[22] Szajewska H., Ruszczynski M. (2010). Systematic review demonstrating that breakfast consumption influences body weight outcomes in children and adolescents in Europe. Crit. Rev. Food Sci. Nutr..

[23] World Health Organization (2003). Diet, Nutrition and the Prevention of Chronic Diseases. http://www.who.int/dietphysicalactivity/publications/trs916/download/en/.

[24] McNaughton S.A., Ball K., Mishra G.D., Crawford D.A. (2008). Dietary patterns of adolescents and risk of obesity and hypertension. J. Nutr..

[25] Avery A., Bostock L., McCullough F. (2015). A systematic review investigating interventions that can help reduce consumption of sugar-sweetened beverages in children leading to changes in body fatness. J. Hum. Nutr. Diet..

[26] Currie C., Roberts C., Morgan A., Smith R., Settertobulte W., Samdal O., Barnekow Rasmussen V. (2004). Young People’s Health in Context. Health Behaviour in School-Aged Children (HBSC) Study: International Report From the 2001/2002 Survey.

[27] Currie C., Gabhainn S.N., Godeau E., Roberts C., Smith R., Currie D., Picket W., Richter M., Morgan A., Barnekow V. (2008). Inequalities in Young People’s Health. Health Behaviour in School-Aged Children International Report from the 2005/2006 Survey.

[28] Yngve A., Wolf A., Poortvliet E., Elmadfa I., Brug J., Ehrenblad B., Franchini B., Haraldsdottir J., Krolner R., Maes L. (2005). Fruit and vegetable intake in a sample of 11-year-old children in 9 European countries: The pro children cross-sectional survey. Ann. Nutr. Metab..

[29] Vereecken C., Pedersen T.P., Ojala K., Krolner R., Dzielska A., Ahluwalia N., Giacchi M., Kelly C. (2015). Fruit and vegetable consumption trends among adolescents from 2002 to 2010 in 33 countries. Eur. J. Public Health.

[30] Lien N., van Stralen M.M., Androutsos O., Bere E., Fernandez-Alvira J.M., Jan N., Kovacs E., van Lippevelde W., Manios Y., te Velde S.J. (2014). The school nutrition environment and its association with soft drink intakes in seven countries across Europe—The energy project. Health Place.

[31] Alexy U., Wicher M., Kersting M. (2010). Breakfast trends in children and adolescents: Frequency and quality. Public Health Nutr..

[32] Elinder L.S., Heinemans N., Zeebari Z., Patterson E. (2014). Longitudinal changes in health behaviours and body weight among Swedish school children—Associations with age, gender and parental education—The scip school cohort. BMC Public Health.

[33] Levin K.A., Kirby J., Currie C., Inchley J. (2012). Trends in adolescent eating behaviour: A multilevel cross-sectional study of 11–15 year olds in Scotland, 2002–2010. J. Public Health.

[34] Stea T.H., Overby N.C., Klepp K.I., Bere E. (2012). Changes in beverage consumption in Norwegian children from 2001 to 2008. Public Health Nutr..

[35] Slining M.M., Mathias K.C., Popkin B.M. (2013). Trends in food and beverage sources among us children and adolescents: 1989–2010. J. Acad. Nutr. Diet..

[36] Fischer C., Brug J., Tak N.I., Yngve A., te Velde S.J. (2011). Differences in fruit and vegetable intake and their determinants among 11-year-old schoolchildren between 2003 and 2009. Int. J. Behav. Nutr. Phys. Act..

[37] Kerr M.A., Rennie K.L., McCaffrey T.A., Wallace J.M., Hannon-Fletcher M.P., Livingstone M.B. (2009). Snacking patterns among adolescents: A comparison of type, frequency and portion size between Britain in 1997 and Northern Ireland in 2005. Br. J. Nutr..

[38] Eilat-Adar S., Koren-Morag N., Siman-Tov M., Livne I., Altmen H. (2011). School-based intervention to promote eating daily and healthy breakfast: A survey and a case-control study. Eur. J. Clin. Nutr..

[39] Levin K.A., Kirby J. (2012). Irregular breakfast consumption in adolescence and the family environment: Underlying causes by family structure. Appetite.

[40] Siega-Riz A.M., Popkin B.M., Carson T. (1998). Trends in breakfast consumption for children in the United States from 1965–1991. Am. J. Clin. Nutr..

[41] Levin K.A., Kirby J., Currie C. (2012). Family structure and breakfast consumption of 11–15 year old boys and girls in Scotland, 1994–2010: A repeated cross-sectional study. BMC Public Health.

[42] Roberts C., Freeman J., Samdal O., Schnohr C.W., de Looze M.E., Nic Gabhainn S., Iannotti R., Rasmussen M. (2009). The health behaviour in school-aged children (HBSC) study: Methodological developments and current tensions. Int. J. Public Health.

[43] Sigmund E., Sigmundova D., Badura P., Kalman M., Hamrik Z., Pavelka J. (2015). Temporal trends in overweight and obesity, physical activity and screen time among Czech adolescents from 2002 to 2014: A national health behaviour in school-aged children study. Int. J. Environ. Res. Public Health.

[44] Health Behaviour in School-aged Children. www.hbsc.org.

[45] Inchley J., Todd J., Bryce C., Currie C. (2001). Dietary trends among Scottish schoolchildren in the 1990s. J. Hum. Nutr. Diet..

[46] Humenikova Shriver L., Gates G. (2009). A cross-cultural comparison of dietary intakes and physical activity between American and Czech school-aged children. Public Health Nutr..

[47] Jakubikova M., Dofkova M., Ruprich J. (2011). Fruit and vegetable intake in the Czech child population. Public Health Nutr..

[48] Bere E., Hilsen M., Klepp K.I. (2010). Effect of the nationwide free school fruit scheme in Norway. Br. J. Nutr..

[49] Bere E., Veierod M.B., Skare O., Klepp K.I. (2007). Free school fruit—Sustained effect three years later. Int. J. Behav. Nutr. Phys. Act..

[50] Hilsen M., van Stralen M.M., Klepp K.I., Bere E. (2011). Changes in 10–12 year old’s fruit and vegetable intake in Norway from 2001 to 2008 in relation to gender and socioeconomic status—A comparison of two cross-sectional groups. Int. J. behav. Nutr. Phys. Act..

[51] Defeyter M.A., Graham P.L., Walton J., Apicella T. (2010). Breakfast clubs: Availability for British schoolchildren and the nutritional, social and academic benefits. Nutr. Bull..

[52] Friedman B.J., Hurd-Crixell S.L. (1999). Nutrient intake of children eating school breakfast. J. Am. Diet. Assoc..

[53] Mhurchu C.N., Gorton D., Turley M., Jiang Y., Michie J., Maddison R., Hattie J. (2013). Effects of a free school breakfast programme on children’s attendance, academic achievement and short-term hunger: Results from a stepped-wedge, cluster randomised controlled trial. J. Epidemiol. Community Health.

[54] Moore L., Moore G.F., Tapper K., Lynch R., Desousa C., Hale J., Roberts C., Murphy S. (2007). Free breakfasts in schools: Design and conduct of a cluster randomised controlled trial of the primary school free breakfast initiative in Wales [ISRCTN18336527]. BMC Public Health.

[55] Evans C.E., Christian M.S., Cleghorn C.L., Greenwood D.C., Cade J.E. (2012). Systematic review and meta-analysis of school-based interventions to improve daily fruit and vegetable intake in children aged 5 to 12 y. Am. J. Clin. Nutr..

[56] Ministerstvo Školství Mládeže a Tělovýchovy (Ministry of Education) Konsolidovaný Text Školského Zákona a Doprovodný Materiál. http://www.msmt.cz/dokumenty/konsolidovany-text-skolskeho-zakona.

[57] Zdravá Strava Do Škol (Healthy Food to School). http://www.zdravastravadoskol.cz.

[58] Skutečně Zdravá Škola (Healthy School). http://www.skutecnezdravaskola.cz.

[59] Happy Snack—Školní Automat Na Zdravou Svačinku (Happy Snack-Vending Machines for Schools). http://www.zdravastravadoskol.cz.

[60] NÚV Pohyb a Výživa (Physical Activity and Nutrition). http://pav.rvp.cz.

[61] SZIF Ovoce a Zelenina Do Škol (Free Fruit and Vegetable at School). http://www.ovocedoskol.szif.cz.

[62] Ransley J.K., Greenwood D.C., Cade J.E., Blenkinsop S., Schagen I., Teeman D., Scott E., White G., Schagen S. (2007). Does the school fruit and vegetable scheme improve children’s diet? A non-randomised controlled trial. J. Epidemiol. Community Health.

[63] Vereecken C., Maes L. (2003). A Belgian study on the reliability and relative validity of the health behaviour in school-aged children food-frequency questionnaire. Public Health Nutr..

[64] Pedersen T.P., Holstein B.E., Laursen B., Rasmussen M. (2015). Main meal frequency measures in the health behaviour in school-aged children study: Agreement with 7-day 24-h recalls. Int. J. Public Health.

[65] Zaborskis A., Moceviciene R., Iannotti R.J. (2014). The influence of chronological period of data collection on differences in reported dietary intake among school-aged children surveyed in 39 countries. J. Nutr. Educ. Behav..

[66] Cuberek R., El Ansari W., Frömel K., Skalik K., Sigmund E. (2010). A comparison of two motion sensors for the assessment of free-living physical activity of adolescents. Int. J. Environ. Res. Public Health.

